# Time-dependent effects of tumor necrosis factor α on Ca^2+^-dependent secretion in murine small intestinal organoids

**DOI:** 10.3389/fphys.2024.1382238

**Published:** 2024-04-26

**Authors:** Svenja Mareike Pauer, Brigitta Buß, Martin Diener, Jasmin Ballout

**Affiliations:** Institute for Veterinary Physiology and Biochemistry, Justus-Liebig-University Giessen, Giessen, Germany

**Keywords:** intestinal organoids, mouse, tumor necrosis factor alpha, secretion, epithelial barrier, Ca^2+^ signaling

## Abstract

**Background:**

Intestinal organoids are stem cell-derived, 3D “mini-guts” with similar functions as the native intestinal epithelium such as electrolyte transport or establishment of an epithelial barrier. During intestinal inflammation, epithelial functions are dysregulated by proinflammatory cytokines like tumor necrosis factor α (TNFα) and other messengers from the immune system resulting in a loss of electrolytes and water due to an impaired epithelial barrier and higher net secretion.

**Methods:**

A murine small intestinal organoid model was established to study (long-term) effects of TNFα on the intestinal epithelium *in vitro* using live imaging, immunohistochemical staining and qPCR.

**Results:**

TNFα induced apoptosis in intestinal organoids as indicated by an increased number of cells with immunoreactivity for cleaved caspase 3. Furthermore, TNFα exposure led to swelling of the organoids which was inhibited by bumetanide and was concomitant with an upregulation of the bumetanide-sensitive Na^+^-K^+^-2Cl^-^ symporter 1 (NKCC1) as shown by qPCR. Fura-2 imaging experiments revealed time-dependent changes in Ca^2+^ signaling consisting of a rise in the basal cytosolic Ca^2+^ concentration at day 1 and an increase of the carbachol-induced Ca^2+^ response after 3 days TNFα exposure. This was prevented by preincubation with La^3+^, an inhibitor of non-selective cation channels, or by using a Ca^2+^-free buffer indicating an enhancement of the Ca^2+^ influx from the extracellular side by the cytokine. No significant changes in cDNA levels of epithelial barrier proteins could be observed in the presence of TNFα.

**Conclusion:**

Intestinal organoids are a useful tool to study the mechanism underlying the TNFα-induced secretion on enterocytes such as the regulation of NKCC1 expression or the modulation of cellular Ca^2+^ signaling.

## Introduction

Since the development of 3D cultures with organ-like properties, organoids became a useful model to study host-microbe interactions, infectious and genetic diseases, or drug responses (Holloway et al., 2019; Kim et al., 2020). Organoids, e.g., from the intestine, can be developed from stem cells and cultivated over several months in a hydrogel matrix with specific cell culture media containing essential growth factors (Sato et al., 2009; Almeqdadi et al., 2019). In the intestinal epithelium, the main population of stem cells is located at the crypt base, from where they proliferate and migrate toward the top of the villi before undergoing apoptosis after a few days. The stem cells are capable of differentiating into enterocytes, goblet cells, Paneth cells, or enteroendocrine cells and fulfilling their different functions like nutrient and electrolyte transport, immune defense, or endocrine signaling (van der Flier and Clevers, 2009; Noah et al., 2011). This morphological and functional diversity can also be found in intestinal organoids after long-term cultivation (Ootani et al., 2009; Yui et al., 2012; Foulke-Abel et al., 2016). Epithelial transport and barrier properties of intestinal organoids have already been shown in various studies (see, e.g., Zietek et al., 2015; Foulke-Abel et al., 2016).

Under healthy conditions, intestinal ion secretion is dominated by the transport of Cl^−^ into the gut lumen, where various channels and transporters such as CFTR (cystic fibrosis conductance transmembrane regulator) or NKCC1 (Na^+^-K^+^-2Cl^−^ cotransporter 1) are involved (Barrett and Keely, 2000). Chloride secretion in the enterocytes is regulated by second messengers such as cAMP, which is generated by adenylate cyclases, or Ca^2+^. This cation can be released from intracellular stores via inositol-1,4,5-trisphosphate (IP_3_), which is produced by phospholipase C (PLC). The release is followed by an entry from the extracellular space via cation channels (Barrett and Keely, 2000).

Stimulation of these pathways leads to enhanced secretion of mainly Cl^−^ into the gut lumen, followed by passive transport of cations and water (Keely and Barrett, 2022). This can be observed symptomatically as secretory diarrhea caused, e.g., by intestinal inflammation, immune mediators, or proinflammatory cytokines like tumor necrosis factor α (TNFα) (McKay and Baird, 1999; Keely and Barrett, 2022). In the human distal colon, TNFα induces an anion secretion via prostaglandin E_2_ (PGE_2_) which is accompanied by an increase in mucosal permeability, i.e., a disruption of the epithelial barrier (Schmitz et al., 1996; Schmitz et al., 1999). Similar observations were made in the rat distal colon, where TNFα enhanced basal ion secretion, measured as short-circuit current (I_sc_), and increased tissue conductance (Ballout and Diener, 2019). In contrast, under inflammatory conditions *in vivo*, such as in a mitomycin-induced colitis model in rats, a downregulation of the secretory response evoked by Ca^2+^-dependent secretagogues has been reported (Kachur et al., 1995). Subsequent studies using the TNBS (2,4,6-trinitrobenzenesulphonic acid) colitis model revealed that the downregulation of carbachol-induced secretion disappeared after removal of the submucosa, suggesting an involvement of the enteric nervous system (Sánchez de Medina et al., 2002). In a previous study from our lab, we observed that in the intestine from TNBS-treated rats, Ca^2+^-dependent (but not cAMP-mediated) Cl^−^ secretion was altered, i.e., the decay of the carbachol-induced I_sc_ was accelerated in the distal colon, but prolonged in the jejunum (Becker et al., 2019). Due to this obvious discrepancy, i.e., short-term stimulation of epithelial secretion by TNFα, but downregulation in chronic *in vivo* models of colitis and contradictory findings in Ca^2+^ signaling, it seemed to be of interest to study long-term effects of proinflammatory cytokines like TNFα on epithelial functions and signaling in an organoid model. This allows for more prolonged cytokine exposure in comparison to classical Ussing chamber experiments or experiments with isolated colonic crypts due to their limited viability. Therefore, we established an organoid model from murine small intestinal stem cells in our lab to study such TNFα-mediated effects on the intestinal epithelium focusing on functional assays such as live imaging (Ca^2+^ imaging and swelling experiments), in combination with immunohistochemistry and qPCR.

## Material and methods

### Animals

For the development of the intestinal organoid cultures, small intestinal tissues from at least 3 C57BL6/J mice with the age of 5–9 days from both sexes were pooled for stem cell isolation. Overall, 3 independent organoid cultures from 11 animals were used for the experiments described in the present study. The animals were bred and housed at the Institute for Veterinary Physiology and Biochemistry of the Justus Liebig University Giessen at an ambient temperature of 23°C–25°C and air humidity of 50%–55% on a 12:12 h light-dark cycle with free access to water and food. Animals were killed by decapitation. Experiments were approved by the named animal welfare officers of the Justus Liebig University (administrative number: 679_M) and performed according to the German and European animal welfare law.

### Solutions

For preparation of intestinal stem cells, 100 mM phosphate buffered saline (PBS) was used; for stem cell isolation 2 mM EDTA (ethylenediaminetetraacetic acid) was dissolved in PBS (EDTA/PBS). Organoid growth medium (IntestiCult™ Organoid Growth Medium Mouse) was obtained from StemCell Technologies (Vancouver, Canada).

Tyrode solution, which was used for Ca^2+^ imaging experiments, consisted of (in mM): 140 NaCl, 5.4 KCl, 10 HEPES [N-(2-hydroxyethyl)piperazine-N′-2-ethanesulfonic acid], 12.2 glucose, 1.25 CaCl_2_, and 1 MgCl_2_. For Ca^2+^ imaging experiments with Ca^2+^-free Tyrode solution, CaCl_2_ was omitted. The immunofluorescence experiments were carried out in phosphate buffer (PB) containing 80 mM Na_2_HPO_4_ and 20 mM NaH_2_PO_4_. For Tyrode solution and PB the pH was set to 7.4 with 1 N HCl/NaOH.

### Intestinal organoid culture

For preparation of intestinal organoids, a modified protocol was used based on the protocols of Sato et al. (2009) and StemCell Technologies (StemCell Technologies, 2016). For one organoid culture, the small intestines from 3–5 neonatal mice were dissected, opened longitudinally and cut into 2 × 2 mm squares followed by several washing steps with ice-cold PBS (30 s sinking time of the fragments, discarding the supernatant and refilling with 10 mL ice-cold PBS) until the supernatant became clear. Ice-cold EDTA/PBS solution was used to isolate crypts (shaking on a hinged plate at 40 rpm for 20–30 min on ice until a sufficient number of single cells and crypt fragments, including stem cells, was observed).

Under sterile conditions, the EDTA/PBS was exchanged with 10 mL ice-cold PBS containing 0.1% (w/v) bovine serum albumin (BSA) (BSA/PBS) and pipetted up and down three times. After 30 s, the cell-containing supernatant was transferred into a 50 mL tube through a 70 μm cell strainer. This step was repeated with the origin pellet up to four times until a purified cell suspension was obtained. The fraction with the highest number of cells/crypt fragments (and a low contamination by cell debris) was selected, centrifuged (5 min at 280 g), mixed with 10 mL ice-cold BSA/PBS, and transferred into a fresh 15 mL tube before it was centrifuged again (5 min at 280 g). The supernatant was removed and the pellet was resuspended with 150 µL IntestiCult™ medium (tempered at 15°C–20°C, StemCell Technologies). The same volume of ice-cold Matrigel^®^ (#356231, growth-factor reduced, Corning, New York, United States) was added to the cell suspension and mixed carefully. 50 μL of the cell/Matrigel^®^ suspension were transferred into the center of a preheated 24-well plate and put into an incubator (37°C, 5% (v/v) CO_2_) for approximately 30 min before each well was covered with 500 µL IntestiCult™ medium (37°C, StemCell Technologies). Medium was exchanged every 2–3 days.

For passaging of intestinal organoids, the medium was removed, the Matrigel^®^ drop was liquefied with ice-cold BSA/PBS and the organoids were disrupted mechanically by up and down pipetting. The cell suspension was transferred into a 15 mL tube and centrifuged at 200 g for 5 min. The pellet was resuspended with fresh IntestiCult™ medium (StemCell Technologies), the same volume of Matrigel^®^ was added and cells were seeded on plates as described above.

To measure the effects of the proinflammatory cytokine TNFα on intestinal organoids, the organoids were incubated with TNFα (100 ng/mL) for 1, 3 or 5 days. Untreated controls were handled in parallel for all experimental series.

### Live imaging of intestinal organoids

Due to the findings that intestinal organoids start changing their morphology after treatment with TNFα (see *Results*), a long-term record over a period of 24 h was performed. The morphological changes were recorded with an Olympus IX81 microscope equipped with a MT illumination system, an Olympus FireWire camera (V2.1.30), and a climatic chamber (Evotec AUC04/B). Live imaging experiments with organoids covered with IntestiCult™ medium in a 24-well plate were run under the following conditions: 37°C, 5% (v/v) CO_2_ and 50% air humidity. 100 ng/mL TNFα was added at the beginning of each experiment. The cAMP-dependent secretagogue forskolin (9 µM) was administered as viability control at the end of each experiment. Every 5 min a bright-field microscopical image was taken and transferred into a video file with 12 frames per second (Olympus xcellence rt software, version 2.0.1). In order to quantify the time course of TNFα-induced changes in morphology, relative lumen size, expressed as luminal area in % of the total organoid’s area, was analyzed every hour using ImageJ (1.53e, W. Rasband and contributors, National Institutes of Health, United States).

To elucidate the underlying mechanisms of TNFα-induced organoid swelling, changes in the relative lumen size of organoids were determined after pretreatment with TNFα (100 ng/mL) + bumetanide (100 μM, a blocker of NKCC), TNFα + indometacin [100 μM, a cyclooxygenase (COX) inhibitor], or TNFα + ethanol [0.5% (v/v), used as solvent of both drugs]. Drugs were administered to the medium and organoids were kept at 37°C and 5% (v/v) CO_2_ for 24 h. Pictures were taken with a light microscope (Nikon ECLIPSE Ts2R, HAMAMATSU ORCA-spark) before and 24 h after the respective treatment.

### Ca^2+^ imaging experiments

For Ca^2+^ imaging experiments (carried out in the same setup used for the live imaging experiments), Matrigel^®^ was removed by gently pipetting with ice-cold BSA/PBS. Organoids were collected and centrifuged for 1 min at 1,000 rpm before 30 µL of the cell suspension was transferred to a glass bottom microwell dish (MatTek Corporation, United States) covered with poly-L-lysin (0.1 mg/L, 70,000–150,000 kDa, Cell Systems, Troisdorf, Germany). Untreated controls or TNFα-treated organoids (100 ng/mL; for 1, 3 or 5 days, respectively) were loaded at room temperature with the membrane permeable form of fura-2, fura-2 acetoxymethylester (fura-2/AM, 6 µM), combined with an equal volume of the nonionic detergent pluronic acid [20% (w/v) stock solution in DMSO] for 60 min. After washing with 500 µL Tyrode solution, the organoids were covered with 4 mL Tyrode solution (either with or without CaCl_2_) and transferred to the imaging setup. The cells were excited alternately with 340 nm or 380 nm and the emission ratio was calculated (340 nm/380 nm) in different regions of interest (ROI), each with the size of one individual cell. An increase in the fura-2 ratio represents an increase in the cytosolic Ca^2+^ concentration.

After a stabilization phase of 3 min to measure the basal fura-2 ratio, the cells were stimulated with the cholinergic agonist carbachol (50 µM). In some experiments La^3+^ (1 mM), a blocker of non-selective cation channels, was administered previous to carbachol. Viability control was set at the end of each experiment by administration of cyclopiazonic acid (CPA, 5 µM), a blocker of the sarcoplasmic-endoplasmic reticulum Ca^2+^ ATPase (SERCA). A response to the respective drug was accepted when two conditions were fulfilled simultaneously: 1.) the amplitude of the change exceeded the 4-fold standard deviation of the scattering in the fura-2 ratio during the control period just prior to addition of the drug. 2.) The amplitude of the change in the fura-2 ratio exceeded an absolute value of 0.1. Cells which did not respond to carbachol or CPA were excluded from further analysis.

### Immunofluorescence

For immunofluorescence experiments, intestinal organoids embedded in Matrigel^®^ were fixed with preheated 4% (w/v) paraformaldehyde (PFA, 37°C) in PBS at room temperature for 30 min. After removing of PFA and rinsing with PBS, the organoids were suspended in BSA/PBS and collected in a 15 mL tube. The supernatant was removed after 15 min and a remaining amount of approximately 1 mL suspension including the organoids was transferred into a 1.5 mL tube, which was shortly spun down for 1 s with a microlitre centrifuge (Roth, Karlsruhe, Germany). The supernatant was removed, the pellet was resuspended with 300 µL liquid gelatin (100 g/L) and transferred into a plastic form for 30 min at 4°C to solidify before it was embedded with tissue freezing medium (Leica Biosystems, Wetzlar, Germany) and cryofixed in N_2_-cooled isopentane. 4 μm thick sections were mounted on microscope slides (Superfrost^®^ Plus, Thermo Fisher Scientific). After rehydration in PB, the sections were incubated for 2 h in a blocking solution containing PB with 0.2% (v/v) triton-X-100, 3% (w/v) BSA, and 10% (v/v) donkey serum. Each primary antibody was dissolved in PB containing 0.1% (v/v) Triton-X-100, 1% (w/v) BSA, 0.5% (w/v) milk powder, and 1% (v/v) donkey serum. After incubation of the sections overnight at 4°C (for sources and final dilutions, see Table 1), the slices were rinsed in PB three times for 5 min each and loaded with the respective secondary antibody for 1 h at room temperature (for combinations see the legend of the respective figure). For nucleus staining and embedding, RotiFluo with DAPI (4,6-diamidio-2-phenylindoldilactate; Roth) was used. Negative staining was performed in parallel without the respective primary antibody. Pictures were taken with the fluorescence microscope Nikon 80i (Nikon, Düsseldorf, Germany). For quantitative analysis, cell counting (e.g., cells with immunoreactivity for cleaved caspase 3) was carried out in a blinded fashion.

**TABLE 1:** Antibodies used for immunofluorescence.

Target	Host	Supplier	RRID	Dilution
*Primary antibodies*				
Cleaved caspase 3	Rabbit	Cell Signaling Technology (9661)	RRID:AB_2341188	1:500
Claudin-3	Rabbit	Invitrogen (34–1700)	RRID:AB_2533158	1:250
NKCC1	Rabbit	Proteintech (13884-1-AP)	RRID:AB_2188522	1:500
Villin	Mouse	Santa Cruz (sc-58897)	RRID:AB_2304475	1:100
ZO-1	Rat	Santa Cruz (sc-33725)	RRID:AB_628459	1:100
*Secondary antibodies*				
Alexa 488 rabbit IgG	Goat	Invitrogen (A-11008)	RRID:AB_143165	1:250
Alexa 488 mouse IgG	Donkey	Invitrogen (A-21202)	RRID:AB_141607	1:250
Cy3 rat IgG	Donkey	Jackson Immuno Research (712-165-150)	RRID:AB_2340666	1:500
Cy3 rabbit IgG	Donkey	Jackson Immuno Research (711-165-152)	RRID:AB_2307443	1:1000

RRID: research resource identifier.

### Histological staining

For histological staining, intestinal organoids were fixed with the same protocol as used for immunohistochemistry, sliced in 4 µm thick sections and rehydrated in PB three times for 5 min each. For hematoxylin-eosin (HE) staining the slides were stained in Mayer’s hemalum for 7 min, followed by rinsing in running tap water for further 7 min and staining with 0.1% (v/v) eosin [addition of 0.01% (v/v) glacial acetic acid] for 5 min. For Periodic Acid Schiff (PAS) staining, the rehydrated slides were transferred into 1% (w/v) periodic acid solution for 10 min, followed by rinsing in tap water for 10 min and in distilled water for 2 min twice. Further staining with Schiff’s reagent for 15 min was followed by washing in 35°C warm tap water before the slides were stained with Mayer’s hemalum for 5 min and rinsed in running tap water for 10 min. After HE and PAS staining, the slides were transferred into staining dishes with increasing ethanol concentrations [70% < 80% < 96%, (v/v)] for a few seconds each. Finally, the slides were treated with Roti-Histol for 3 min twice and mounted with Roti-Histokit. All staining reagents were obtained from Carl Roth GmbH, Karlsruhe, Germany. Pictures were taken with the Nikon 80i light microscope.

### qPCR

For qPCR experiments, Matrigel^®^ was liquified with ice-cold BSA/PBS. The organoid suspension was centrifuged for 5 min at 280 g and supernatant was discarded. The pellet was resuspended with lysis buffer (Macherey-Nagel, Düren, Germany), homogenized with a mixer mill (for 2 min at 30 Hz), and the total RNA was extracted by using the RNA Plus kit including columns to remove genomic DNA (Macherey-Nagel). The concentration of RNA (ng/µL) in each sample and their purity (OD260/280) was determined with the Nanodrop One^©^ (Thermo Fisher Scientific). An equal amount of RNA was transcribed to cDNA with the High-Capacity RNA-to-cDNA Kit (Thermo Fisher Scientific). The qPCR experiments were performed with TaqMan^®^ Gene Expression Master Mix (Thermo Fisher Scientific). All qPCR steps were performed in accordance to manufactures’ instructions.

Overall, seven reference genes were selected either from Thermo Fisher Scientific [glyceraldehyde-3-phosphate dehydrogenase (Gapdh, Mm99999915_g1); ribosomal protein lateral stalk subunit P0 (Rplp0), Mm00725448_s1; eukaryotic translation elongation factor 2 (Eef2), Mm05700170_g1; β-glucuronidase (Gusb), Mm01197698_m1] or from PrimerDesign geNorm 12 gene kit mouse (mitochondrial ATP synthase (ATP5B) and 18s; Z-ge-SY-12-month). Gapdh and Gusb were chosen as the most stable reference genes expressed in the control and TNFα-treated organoids after analysis of inter- and intragroup variances with NormFinder (MOMA, Denmark; Andersen et al., 2004).

Final primers used for qPCR were obtained from Thermo Fisher Scientific (for detailed information see Table 2). For each primer, 3 technical replicates and at least 3 biological replicates were used (3 independent organoid passages), and each qPCR protocol was run twice. The individual efficiency for each amplicon was calculated with LinRegPCR (http://www.hartfaalcentrum.nl). A mean efficiency per amplicon group was determined and outliers (more than 5% of the mean) were excluded. The baseline corrected C_t_ values were used for comparison of expression levels with the efficiency-corrected ΔC_t_ method.

**TABLE 2:** Primers used for qPCR.

Target (gene name)	Assay ID	Gene number
Gapdh	Mm99999915_g1	NM_008084.3
Gusb	Mm01197698_m1	NM_010368.1
Claudin-2 (Cldn-2)	Mm00516703_s1	NM_016675.4
Claudin-3 (Cldn-3)	Mm00515499_s1	NM_009902.4
ZO-1 (Tjp1)	Mm01320638_m1	NM_001163574.1
CFTR	Mm00445197_m1	NM_021050.2
NKCC1 (SLC12A2)	Mm01265951_m1	NM_009194.3
Muc2	Mm01276676_m1	NM_023566.3
Iκbα (Nfkbia)	Mm00477798_m1	NM_010907.2

Gene numbers refer to http://www.ncbi.nlm.nih.gov.

### Drugs

Carbachol and LaCl_3_ were dissolved in aqueous stock solutions. Forskolin, bumetanide, and indometacin were dissolved in ethanol [final maximal ethanol concentration 0.5% (v/v)]. Dimethyl sulfoxide (DMSO) was used as solvent for cyclopiazonic acid [CPA; maximal DMSO concentration 0.1% (v/v)]. Recombinant TNFα from mouse (activity 20·10^6^ units/mg) was dissolved in sterile BSA/PBS. All drugs were obtained from Sigma, Taufkirchen, Germany.

### Statistics

In general, results are given as mean ± standard error of the mean (SEM) with number (n) of investigated organoids or cells. Relative expression ratios (Figure 7) are given as mean and 95% confidence interval. Experiments were repeated at least 3 times with 3 independent organoid passages. For comparison of two groups Student’s *t*-test or Mann-Whitney U-test was performed. In order to find out which test method had to be used an F-test was applied. For comparison of more than two groups, analysis of variance (ANOVA) followed by Tukey’s *post hoc* test was used. Linear regression was calculated with GraphPad Prism 5.01 (GraphPad Software, La Jolla, CA, United States). Statistical analysis of C_t_ values was performed with REST^©^ (REST 2009) for group-wise comparison of qPCR data (Pfaffl et al., 2002). *p* < 0.05 was considered to be statistically significant.

## Results

### Morphology of intestinal organoids and expression of epithelial barrier proteins

**FIGURE 1:**
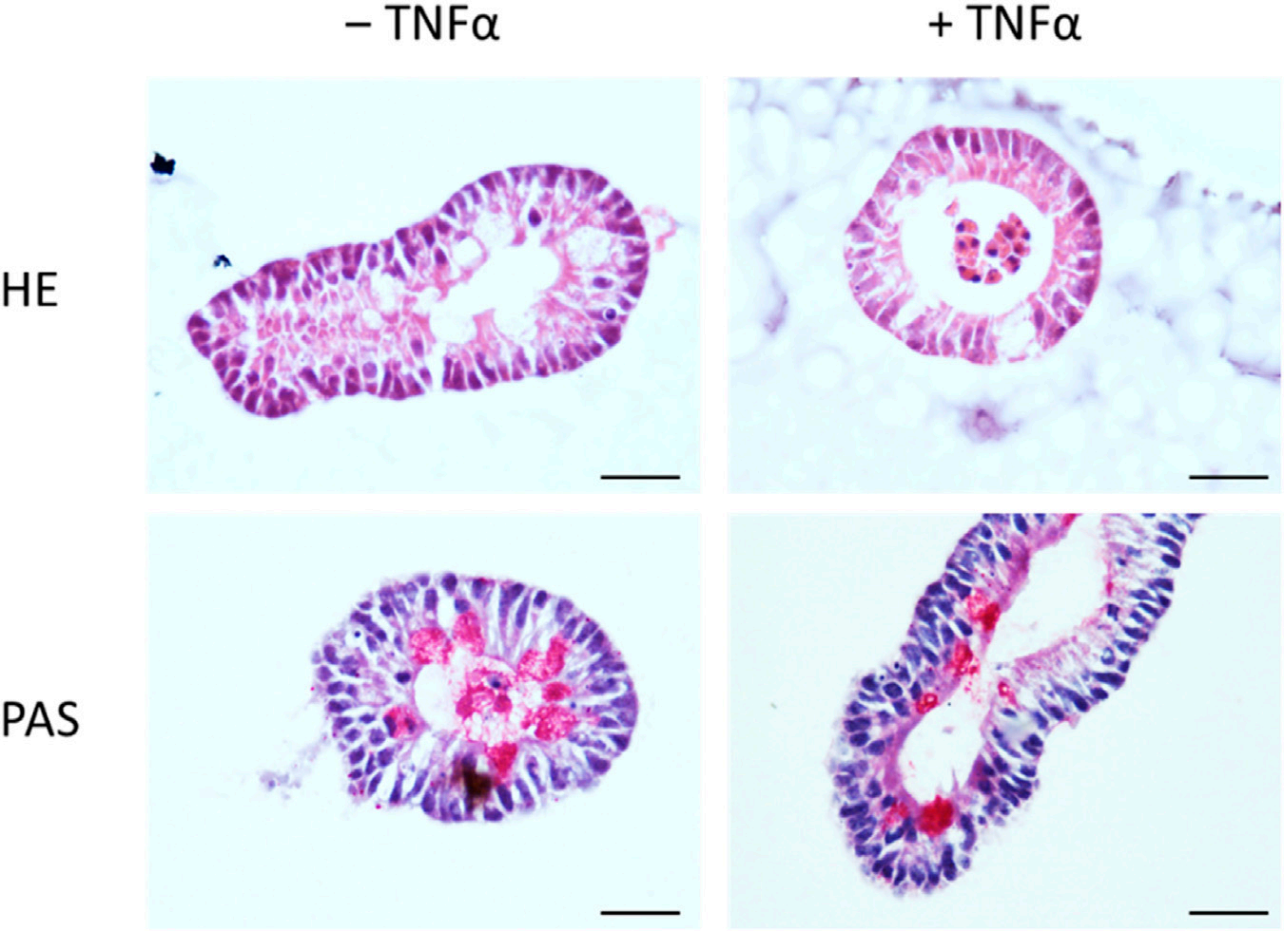
Staining of untreated intestinal organoids (‒ TNFα, left column) or after incubation with 100 ng/mL TNFα for 24 h (+TNFα, right column) with hematoxylin-eosin (HE, upper row) or Periodic Acid Schiff (PAS, lower row) reagent. Scale bar is set to 25 µm. Representative pictures from 3 independent staining with 3 different organoid passages.

**FIGURE 2:**
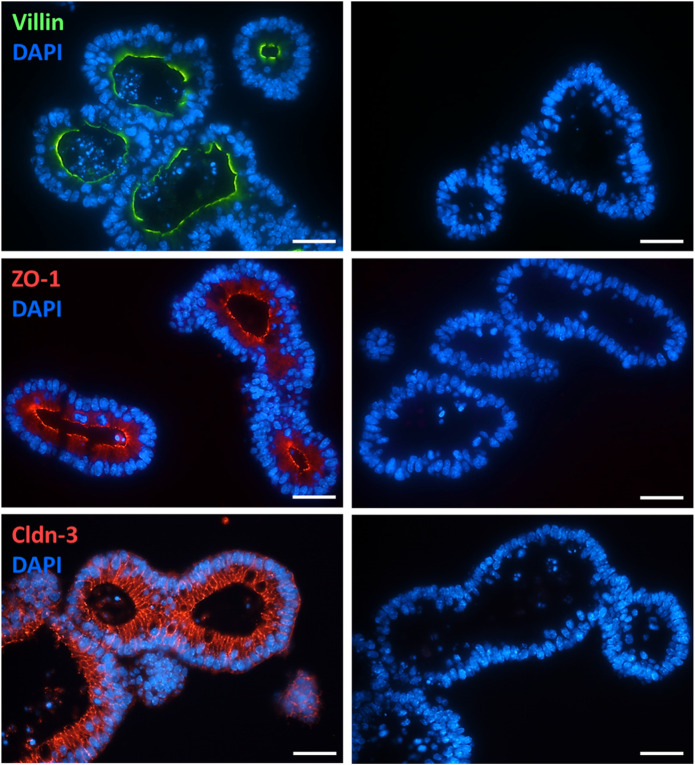
Localization of villin (combined with Alexa488 donkey anti mouse IgG), ZO-1 (combined with Cy3 donkey anti rat IgG), and claudin-3 (Cldn-3, combined with Cy3 donkey anti rabbit IgG) in intestinal organoids (for dilution and supplier see Table 1). Negative staining without the respective primary antibody was performed in parallel (right column). DAPI was used for nucleus staining. Representative staining from 3 independent experiments. Scale bar is set to 20 µm.

Conventional and immunofluorescent stainings were performed to validate the morphology and epithelial barrier function of the intestinal organoid culture. HE-stained organoids revealed the typical morphology of the intestinal epithelium, i.e., columnar enterocytes arranged around a central lumen, whereas PAS staining confirmed the presence of mucus producing goblet cells (Figure 1). The polarity of the epithelial cells in the organoids was confirmed by the distribution of villin, an actin-binding protein in the intestinal brush border, which was found on the apical pole of the cells (Figure 2). The establishment of epithelial cell contacts was confirmed by the expression of zonula occludens 1 (ZO-1), a tight junction-associated scaffolding protein, and claudin-3, a barrier-forming tight junction protein, which were located on the subapical or basolateral side, respectively (Figure 2). Negative staining without the respective primary antibody did not show any signal (Figure 2).

### Morphological changes induced by TNFα

**FIGURE 3:**
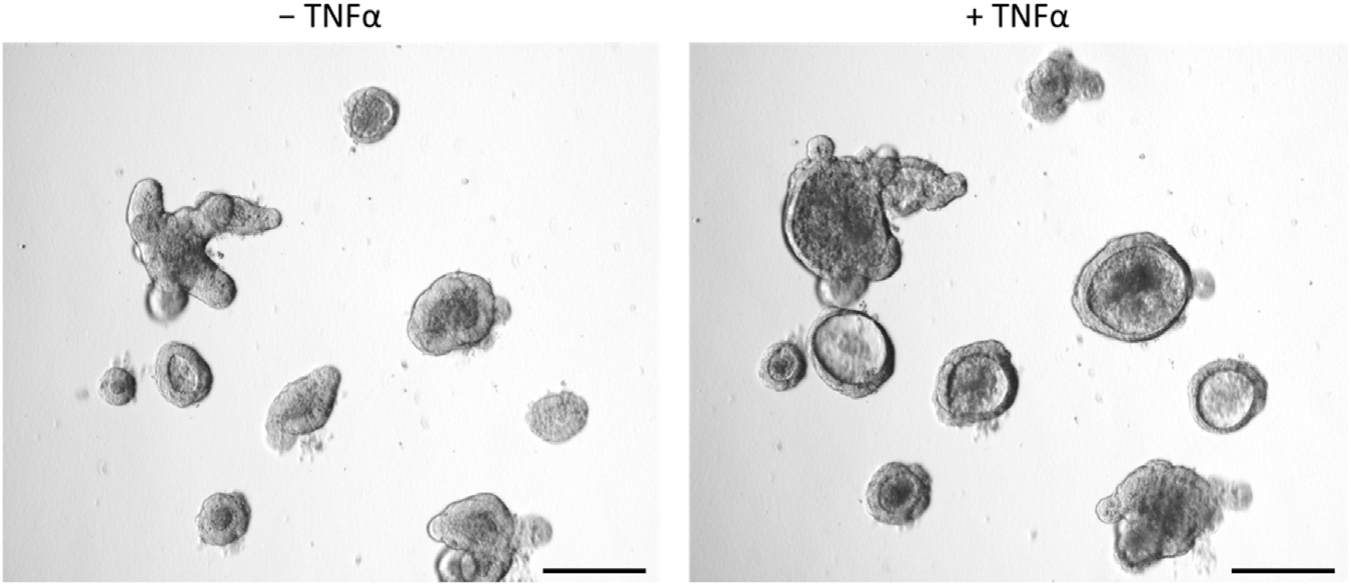
Intestinal organoids before (‒ TNFα, left column) or after incubation with 100 ng/mL TNFα for 24 h (+TNFα, right column). Prolonged incubation (3 days, 5 days) showed similar results. Scale bar is set to 200 µm. Representative pictures from 3 independent organoid passages.

After incubation with TNFα (100 ng/mL, 24 h), the shape of the organoids shifted from the typical branched to a more rounded form with an enlarged lumen filled with fluid, cells or cell fragments (Figure 3). In addition, the epithelium flattened continuously during incubation time (see also time-lapse Supplementary Video S1).

The observed TNFα-induced “organoid swelling” (Figure 3; Supplementary Video S1) could have the following reasons:

1. Passive extension by enhanced luminal cell content due to increased number of apoptotic cells
2. Active secretion of electrolytes and water into the lumen
3. Disrupted epithelial barrier which would facilitate osmotically driven water flux into the organoid’s lumen.

In order to differentiate between these possibilities, immunofluorescent staining, Ca^2+^ imaging, morphometric analyses, and qPCR for expression of tight junctions and Cl^−^ transporters were performed.

### TNFα induced apoptosis in intestinal organoids

**FIGURE 4:**
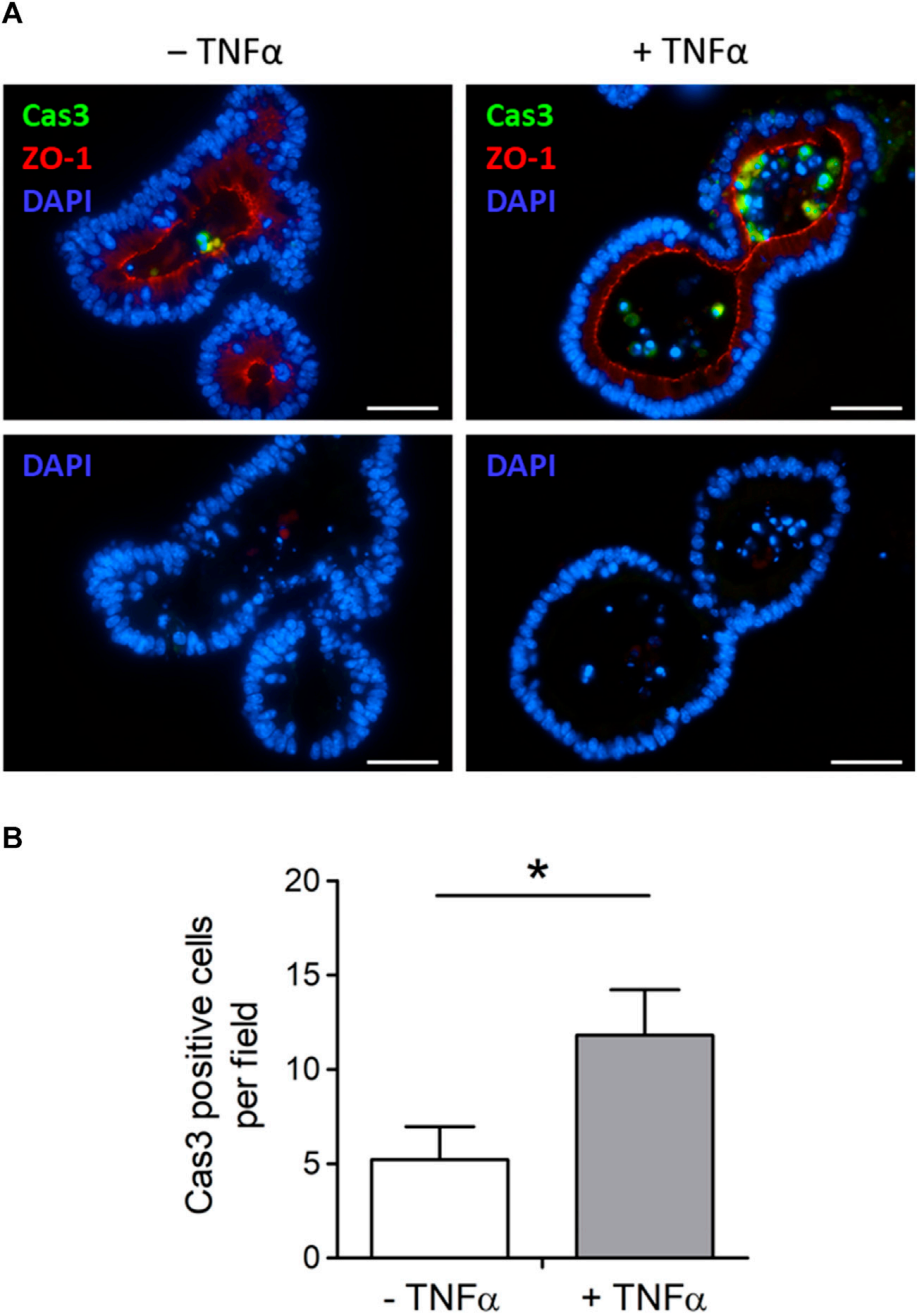
**(A)** Representative immunofluorescent staining from 3 independent experiments of cleaved caspase-3-positive cells (Cas3, combined with Alexa488 goat anti rabbit IgG). Intestinal organoids were either untreated (‒ TNFα, left side) or preincubated with 100 ng/mL TNFα (+TNFα, right side) for 1 day in advance. ZO-1 (combined with Cy3 donkey anti rat IgG) was used as luminal marker and DAPI for nucleus staining. Scale bar is set to 40 µm. **(B)** For blinded analysis only Cas3 and DAPI positive cells were counted per image (*n* = 14–16) from *n* = 22–25 organoids from 3 independent passages. Data are means + SEM and *p < 0.05 was set to be statistically significant.

Tumor necrosis factor α, administered with the same protocol as for the swelling experiments depicted in Figure 3 (100 ng/mL, exposure time 1 day), increased the amount of desquamated material within the lumen of intestinal organoids (Figure 3). To investigate if this is caused by a higher number of apoptotic cells in the lumen, sections from fixed organoids were stained with an antibody against cleaved caspase-3, a key enzyme in the apoptosis cascade. The number of caspase-3 positive cells per image, which also showed a positive nucleus staining with DAPI, was counted (Figure 4). It reached 5.21 ± 1.76 cells per image in the untreated control group (*n* = 14 analyzed images containing a total of 22 organoids) and was more than doubled after 1 day of TNFα incubation (11.81 ± 2.41 cells per image, *n* = 16 analyzed images containing a total of 25 organoids, *p* < 0.05, Figure 4B).

### TNFα increased the relative lumen size of intestinal organoids

**FIGURE 5:**
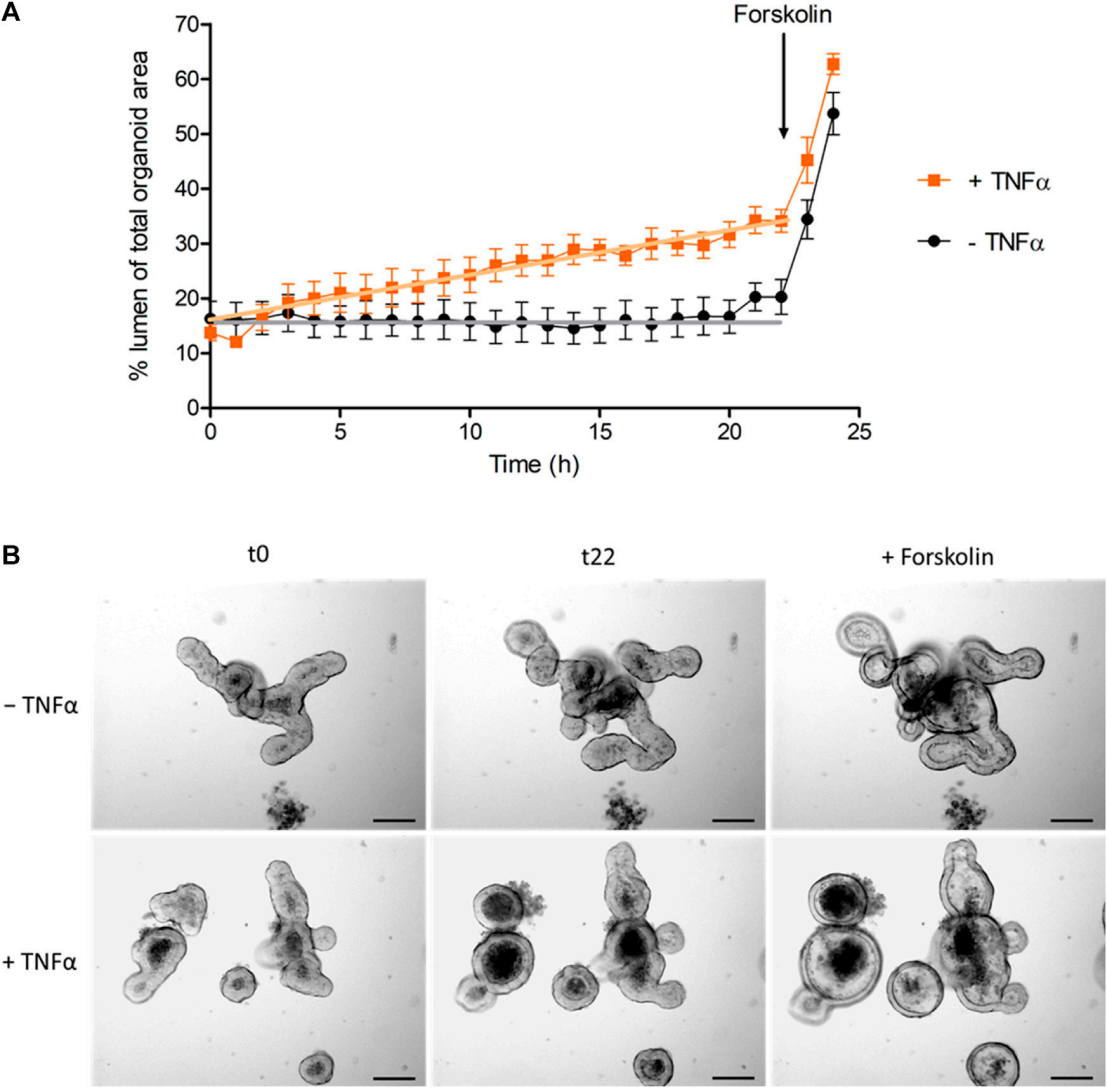
**(A)** Change of luminal organoid size over 24 h under control conditions (‒ TNFα) or after administration of 100 ng/mL TNFα (+TNFα). Forskolin (9 µM) was used as viability control (for statistic of forskolin-induced swelling see results). Data are expressed as % luminal area of the total organoid area and are means ± SEM (for graphical clarity only values per hour are presented) from *n* = 5–7 organoids per group and 3 independent experiments. GraphPad Prism (5.01) was used for linear regression analysis of the time course before administration of forskolin (0–22 h). Statistical analysis shows that the slope of the TNFα-treated group (0.87% ± 0.04%/h) differed significantly from the slope of the control group (0.07% ± 0.04%/h, *p* < 0.05). **(B)** Representative pictures of the swelling experiments [statistic is shown in **(A)**] from untreated (‒ TNFα) and TNFα-treated organoids (100 ng/mL; + TNFα) at initial time point (t0), at 22 h (t22) and after addition of 9 µM forskolin (t24; see also Supplementary Video S1).

Not only the number of apoptotic cells in the lumen was increased after TNFα treatment, but also the relative lumen size of the intestinal organoids. To differentiate if this observation is a direct effect of TNFα, time-lapse video recordings from the intestinal organoids were taken over a period of 24 h under control conditions or in the presence of 100 ng/mL TNFα (Supplementary Video S1). Linear regression analysis exhibited a constant and significant increase in the relative lumen size of TNFα-treated organoids compared to untreated controls (Figure 5). The cAMP-dependent secretagogue forskolin (9 µM) was used as viability control at the end of each experiment (Supplementary Video S1). Forskolin led to an increase of the relative lumen size from 20% to 54% in the control group (*n* = 5 organoids), which did not differ from the increase after 24 h incubation with 100 ng/mL TNFα (from 34% to 63%, *n* = 7 organoids, Figure 5).

**FIGURE 6:**
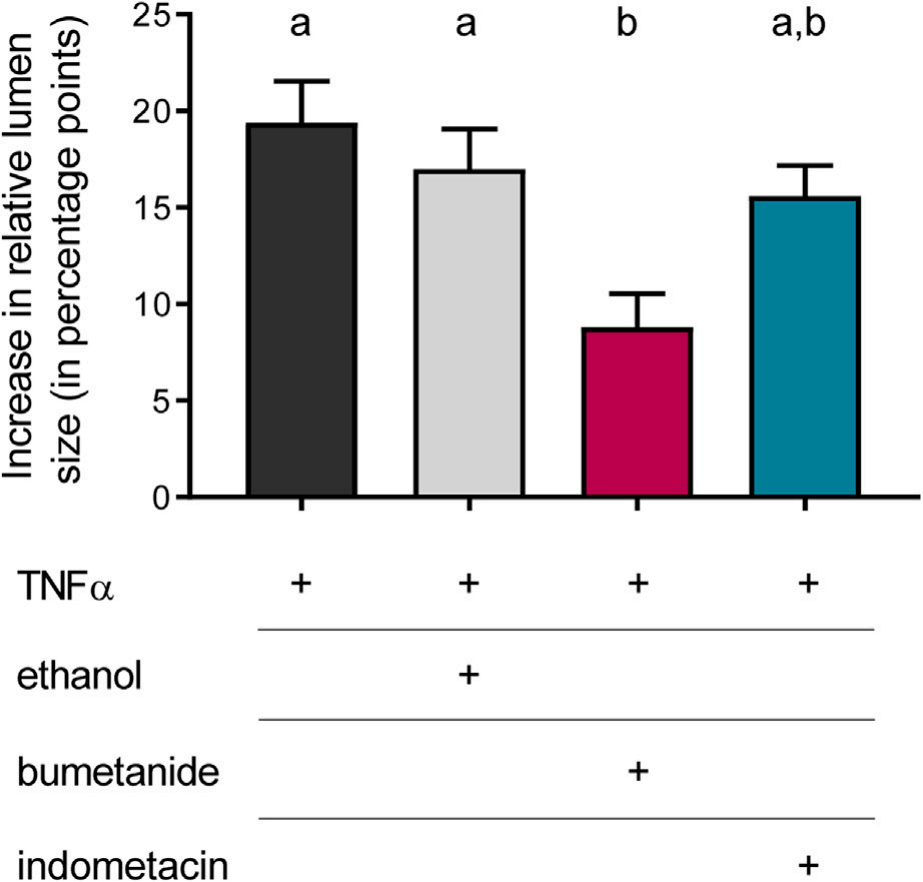
Relative increase in lumen size after 24 h treated with TNFα (100 ng/mL) compared to the increase after incubation with TNFα + bumetanide (100 µM) as well as TNFα + indometacin (100 µM). Ethanol [0.5% (v/v)] was used as solvent control. Data are given as difference in increase of the relative lumen size (i.e., luminal area in % of the total organoid’s area; determined with ImageJ) at the beginning and the end of each experiment and are means + SEM. *n* = 3 passages of intestinal organoids with 10 organoids per group. Statistical homogeneous groups are indicated by the same letter (a, b; *p* < 0.05, ANOVA followed by Tukey’s *post hoc* test).

Inhibitor experiments were carried out to get insights into the mechanisms involved in the TNFα-induced organoid swelling. In the absence of any inhibitors, 100 ng/mL TNFα caused an increase in relative lumen size by 19.4 ± 2.1 percentage points after 24 h incubation (Figure 6). In order to find out whether this swelling was due to Cl^−^ secretion, the organoids were pretreated with bumetanide (100 µM), which blocks the Na^+^-K^+^-2Cl^−^ cotransporter (NKCC1) responsible for basolateral uptake of Cl^−^ to be secreted across the apical membrane. Indeed, TNFα-induced swelling was significantly diminished by about half in the presence of bumetanide (*p* < 0.05). To see if prostaglandins, which are known to mediate TNFα-induced secretion in human colon (Schmitz et al., 1996; Schmitz et al., 1999), might be involved in the TNFα-induced swelling, the COX inhibitor indometacin (100 µM) was administered in combination with TNFα for 24 h. Indometacin as well as ethanol, which was used as solvent control, did not affect the TNFα-induced swelling (Figure 6). Hence, the bumetanide-sensitive NKCC1 seems to be, at least in part, a target of the fluid secretion enhanced by TNFα.

### NKCC1 gene expression is enhanced in TNFα-treated organoids

**FIGURE 7:**
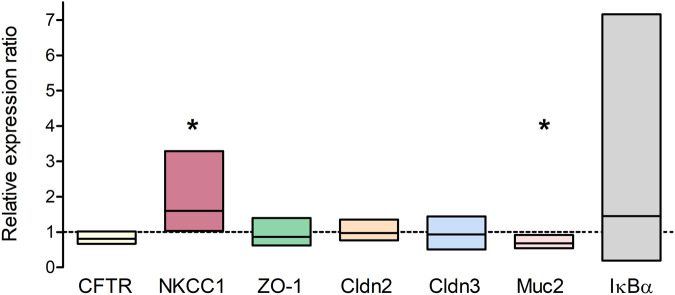
Relative expression ratio of TNFα-treated organoids (100 ng/mL TNFα for 5 days) compared to untreated controls (dotted line at 1) showing either an up- (>1) or downregulation (<1) of the respective target gene. Results were normalized to the reference genes Gapdh and Gusb. Values are arithmetic mean (line within the box) ± 95% confidence interval (box) from *n* = 3 technical and biological replicates (3 independent organoid passages). *p < 0.05 indicates statistical significance (group-wise comparison analyzed with REST 2009, for more information see Methods).

qPCR was performed to analyze long-term effects of TNFα on the expression of different epithelial barrier proteins (ZO-1, claudin-2 and -3) and transporters which are involved in chloride secretion (CFTR, NKCC1). The relative expression in TNFα-treated organoids (100 ng/mL, 5 days) was compared to the expression in untreated controls (*n* = 3 technical and biological replicates, respectively). NKCC1 was significantly higher expressed in TNFα-treated organoids compared to the respective controls, whereas the goblet cell marker mucin 2 (Muc2) was reduced; all other genes showed neither an up- nor a downregulation (Figure 7). The Nfκb inhibitor alpha, Iκbα (Nfκbia), which is increased, e.g., after 48 h TNFα incubation in chromaffin cells (Ait-Ali et al., 2008), served as a positive control for TNFα treatment and tended to be upregulated without reaching statistical significance due to the high variance (Figure 7).

**FIGURE 8:**
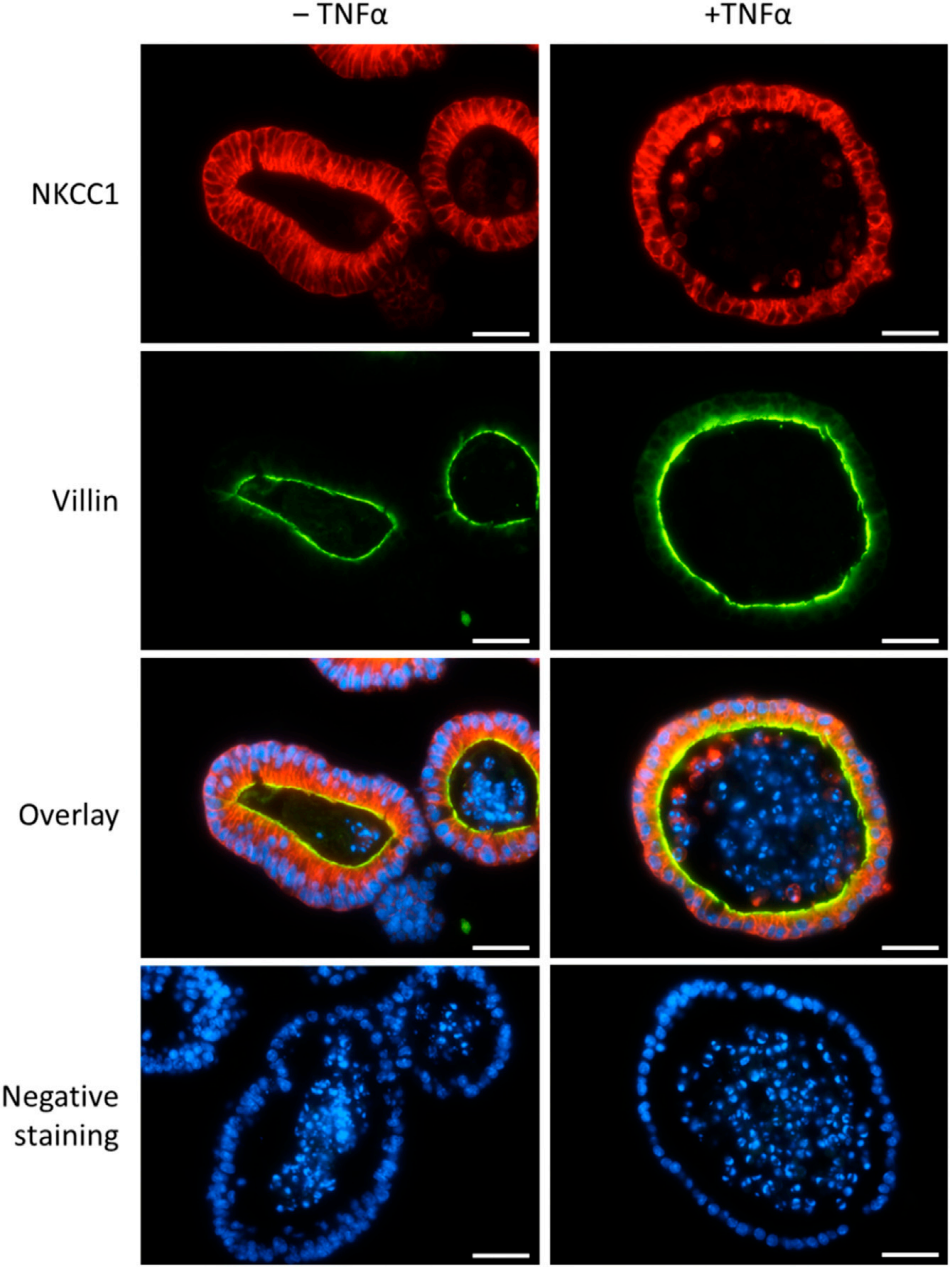
Representative staining from *n* = 3 passages of untreated intestinal organoids (left column) or after TNFα incubation for 1 day (100 ng/mL, right column) with primary antibodies against NKCC1 (1st row, in red, combined with Cy3 donkey anti rabbit) and villin (2nd row, in green, combined with Alexa488 donkey anti mouse). DAPI was used for nucleus staining. Negative controls (last row) without the respective primary antibodies were handled in parallel. Scale bar is set to 25 µm.

In order to confirm the expression of NKCC1 on the protein level, immunofluorescent staining of untreated and TNFα-treated organoids (100 ng/mL, 1 day and 5 days) was performed. As it is typical for the intestinal epithelium, the Na^+^-K^+^-2Cl^-^ cotransporter was expressed on the basolateral membrane of organoids (Figure 8). This pattern did not change in an obvious manner after 1 day (Figure 8) nor after 5 days (data not shown as it was identical to 1 day) of TNFα incubation.

### Ca^2+^ signaling in TNFα-treated intestinal organoids

The second messenger Ca^2+^ is known to be centrally involved in the induction of Cl^−^ secretion, e.g., after stimulation with acetylcholine. Experiments with fura-2 loaded organoids were carried out in order to find out whether TNFα (100 ng/mL) might interfere with this Ca^2+^ signaling. Hence, untreated or TNFα-treated organoids were stimulated with the cholinergic agonist carbachol (50 µM) and the SERCA blocker cyclopiazonic acid (CPA, 5 µM) as viability control. Basal fura-2 ratio changed in dependence on the incubation time with TNFα. After 1 day cytokine exposure, the fura-2 ratio of the organoids, kept in Ca^2+^-containing buffer, was significantly increased compared to the untreated control group (3.55 ± 0.06 versus 3.09 ± 0.07, *p* < 0.05, Table 3) indicating a higher basal cytosolic Ca^2+^ concentration. After 3 days incubation, the difference between the TNFα and the control group was lost and after 5 days the TNFα-treated organoids showed lower basal fura-2 ratios than the control organoids (Table 3). The carbachol-induced increase in the cytosolic Ca^2+^ concentration exhibited a different time pattern: it increased significantly from 1.17 ± 0.14 in the control group to 1.65 ± 0.07 (*p* < 0.05) after 3 days exposure to the cytokine, whereas no significant differences were observed at the other two time points (1 day or 5 days; Table 3). Interestingly, the carbachol-induced rise in fura-2 ratio reached in general higher values with increasing age of organoid culture (Table 3).

**TABLE 3:** Calcium response of intestinal organoids after time-dependent incubation with TNFα.

			Basal fura-2 ratio	Carbachol-induced Δfura-2 ratio	Cells responding to carbachol	
					%	n
Ca^2+^ buffer	1 day	+ TNFα	3.55 ± 0.06*	1.10 ± 0.05	100	50/50
		‒ TNFα	3.09 ± 0.07	1.01 ± 0.05	100	50/50
	3 days	+ TNFα	3.03 ± 0.06	1.65 ± 0.07*	98	60/61
		‒ TNFα	2.98 ± 0.09	1.17 ± 0.14	100	55/55
	5 days	+ TNFα	2.71 ± 0.16*	1.93 ± 0.16	100	51/51
		‒ TNFα	3.22 ± 0.07	1.92 ± 0.13	100	50/50
Ca^2+^-free buffer	1 day	+ TNFα	3.07 ± 0.07*	0.65 ± 0.05^#^	96	55/57
		‒ TNFα	3.30 ± 0.08	0.56 ± 0.04^#^	72	38/53
	3 days	+ TNFα	2.83 ± 0.12	0.92 ± 0.06^#^	85	52/61
		‒ TNFα	2.75 ± 0.08	0.81 ± 0.08^#^	100	53/53
	5 days	+ TNFα	2.04 ± 0.05*	1.24 ± 0.09^#^	93	53/57
		‒ TNFα	3.44 ± 0.13	1.46 ± 0.11^#^	85	40/47

Fura-2 ratio (340 nm/380 nm) of intestinal organoids with or without TNFα (100 ng/mL) pretreatment under basal conditions and increase above baseline induced by carbachol (50 μM, Δfura-2 ratio). Data are means ± SEM. The last two columns give the % and the absolute numbers of cells responding to carbachol (for definition of responders, see *Material and methods*). *p < 0.05 in comparison to the respective untreated group (‒ TNFα). ^#^ *p* < 0.05 carbachol peak compared to respective group in Ca^2+^-containing buffer. In total 626/638 cells reacted to the viability control CPA (5 µM). For one organoid containing 7 ROIs, the CPA response could not be measured due to technical problems.

**FIGURE 9:**
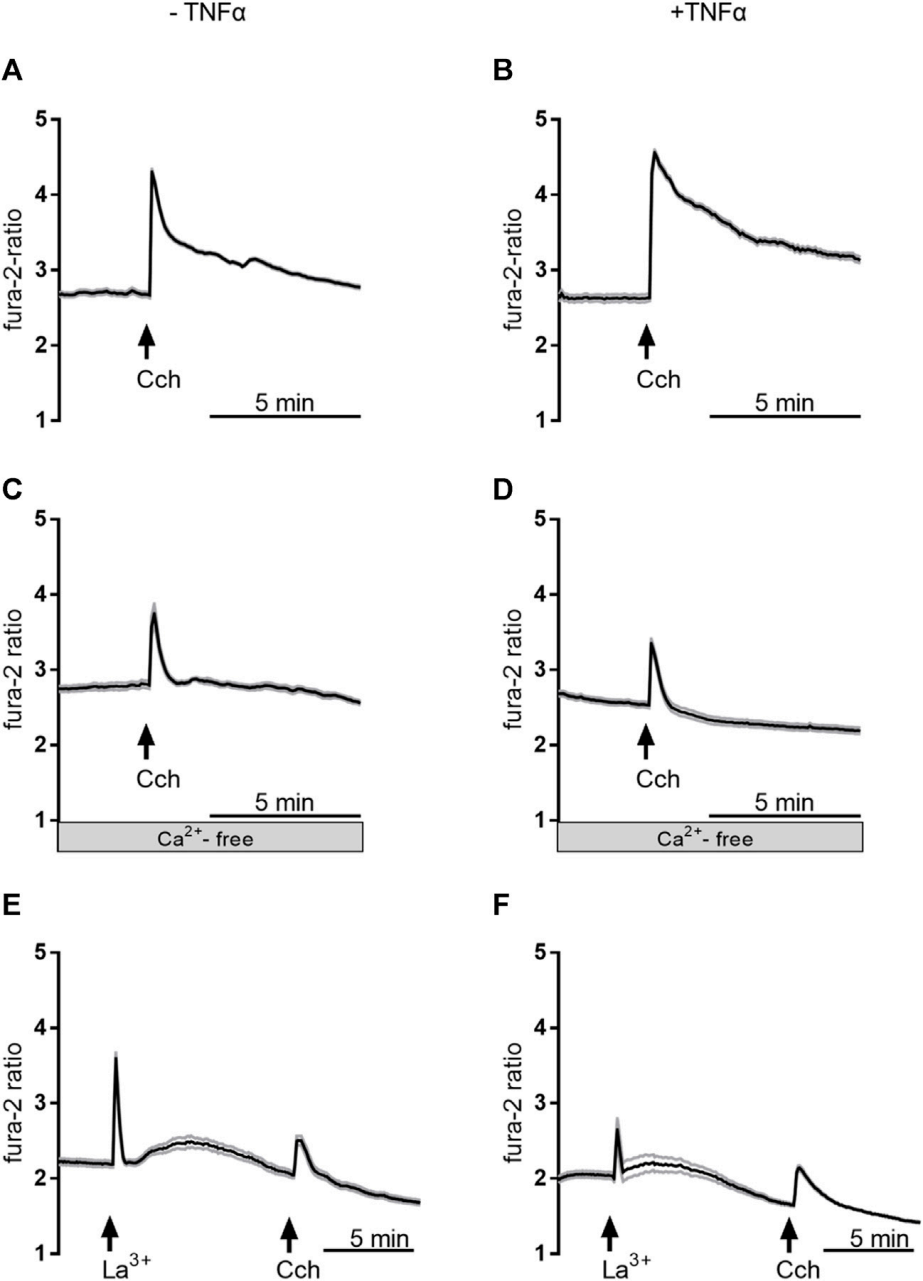
Representative curves of Ca^2+^-imaging experiments demonstrating the effect of carbachol (50 µM) in Ca^2+^-containing buffer in the absence of any inhibitors **(A,B)**, in Ca^2+^-free buffer **(C,D)**, or in Ca^2+^-containing buffer with La^3+^ 1 mM; **(E,F)**. The measurements on the left side **(A,C,E)** were from untreated control organoids, whereas the measurements on the right side **(B,D,F)** were from organoids which were pretreated with TNFα (100 ng/mL) for 3 days. Values are means (thick line) ± SEM (grey lines), *n* = 6–17 cells within one organoid. For statistics, see Tables 3, 4.

In order to find out whether the transient enhancement of the carbachol response by 3 days TNFα incubation might be caused by a stronger Ca^2+^ influx from the extracellular space or by an enhanced release from intracellular stores, experiments with Ca^2+^-free Tyrode solution were performed. Under these conditions, the carbachol-induced Δfura-2 ratio was significantly reduced by more than 40% in comparison to the response in Ca^2+^-containing buffer (Table 3; Figure 9). In the absence of extracellular Ca^2+^, at no time point TNFα was able to enhance the carbachol response significantly above the value of the control group (Table 3).

To investigate if Ca^2+^-permeable cation channels in the cell membrane are responsible for the enhancement of the carbachol-response, experiments with the lanthanide, La^3+^, a broad blocker of non-selective cation channels, were performed in Ca^2+^-containing buffer. Indeed, when La^3+^ (1 mM) was administered to the organoids after 3 days TNFα incubation, the carbachol response was significantly reduced by approximately 20% compared to the untreated controls and only 50% of the cells responded to the stimulation with carbachol (Table 4; Figure 9). Unexpectedly, the lanthanide alone induced a transient increase in the fura-2 signal (see *Discussion*). In conclusion, TNFα seems to have time-dependent effects on Ca^2+^ signaling in intestinal organoids and enhances carbachol-induced secretion via Ca^2+^-permeable cation channels in the membrane.

**TABLE 4:** Calcium response of intestinal organoids to carbachol in the presence of lanthanum.

		Carbachol-induced Δfura-2 ratio	Cells responding to carbachol	
			%	n
Ca^2+^ buffer	+ TNFα	0.36 ± 0.03*	45.2	28/62
	‒ TNFα	0.46 ± 0.03	61.5	32/52

Increase in the fura-2 ratio signal (340 nm/380 nm) induced by carbachol (50 µM) in the presence of La^3+^ (1 mM) with or without 3 days pretreatment with TNFα (100 ng/mL). Data are given as increase in the fura-2 signal above baseline (Δfura-2 ratio) and are means ± SEM. La^3+^ itself induced a transient increase in the fura-2 ratio signal which amounted to 1.22 ± 0.08 in the control group and 1.03 ± 0.11 in the TNFα-pretreated organoids. The last two columns give the % and the absolute numbers of cells responding to carbachol (for definition of responders, see *Material and methods*) *p < 0.05 in comparison to the control group (‒ TNFα).

## Discussion

The pathogenic mechanisms underlying inflammatory bowel diseases (IBD), like Crohn’s disease or colitis ulcerosa, are not fully understood yet. Various genetic, environmental, or pathologic factors lead to a cytokine-driven, chronic inflammation of the gut without an adequate regulation by the intestinal immune system (for detailed reviews see, e.g., Neurath, 2014; Strober et al., 2007; Silva et al., 2016). The most common way to study such pathogenic mechanisms of intestinal inflammation experimentally is the use of epithelial cell lines, like HT-29 or Caco-2, cultivated with different proinflammatory cytokines (Bode et al., 1998; Vallee et al., 2004), or enteritis/colitis models, where, e.g., chemical compounds, like dextran sulfate sodium (DSS) or TNBS, are administered to laboratory animals (Pérez-Navarro et al., 2005; Steidle et al., 2013; Becker et al., 2019). In accordance with the 3R principle, stem cell-derived organoids become more and more a useful tool to study cell functions and interactions in a more physiologically relevant way than can be achieved with epithelial cell lines. Even in the investigation of IBD-based pathomechanisms stem cell-derived organoid cultures from patients are used, but in these circumstances, the focus is predominantly on molecular biological and immunohistochemical approaches without investigating functional properties of the epithelium (Yoo and Donowitz, 2019; Gleeson et al., 2020; Xu et al., 2021).

During intestinal inflammation, elevated proinflammatory cytokine levels [e.g., interleukin (IL)-1β, interferon (IFN)-γ, and TNFα] are found in blood and tissue samples contributing to the vicious cycle of inflammation and inadequate immune responses in the intestinal wall (Pérez-Navarro et al., 2005; Bauer et al., 2010; Neurath, 2014; Singh et al., 2016). However, less is known about how these cytokines interfere directly with the intestinal epithelium regarding, e.g., ion transport and signaling, especially over a longer period. Therefore, we used an organoid model from murine small intestinal stem cells to investigate the (long-term) effects of TNFα on epithelial functions.

A well-known effect of TNFα is the induction of apoptosis via tumor necrosis factor receptor type 1 (TNFR1). Activation of this receptor is followed by an intracellular signal cascade, where different caspases are crucially involved. The activation of caspase-3 is a “point of no return,” from where the programmed cell death can’t be revoked (Aggarwal, 2003). Indeed, the number of caspase-3 positive cells in the lumen of the organoids was strongly increased after incubation with TNFα (Figure 4). A similar finding has already been reported in murine enteroids by Jones et al. (2019), where mice were treated by intraperitoneal injection of 0.33 mg/kg TNFα *in vivo*. Albeit the number of caspase-3 positive cells increased significantly in the small intestine as well as in the enteroids generated from these mice, the number of Ki67 positive cells, a marker for proliferation, was not changed in this study. Hence, the intraluminal cell accumulation observed in our experiments might contribute to a certain extent to TNFα-induced organoid swelling, although a stimulation of net secretion followed by passive influx of water appears to be more relevant (see below).

Possible changes in the barrier properties of the epithelium, which determine the passive flux of water or electrolytes via the intercellular space, were investigated by expression experiments with selected epithelial barrier proteins. Immunohistochemical control experiments with the marker proteins villin, ZO-1, and claudin-3 (Figure 2) suggest a physiological differentiation of the organoids with clear apical and basolateral cell poles. Interestingly, long-term incubation (5 days) with 100 ng/mL TNFα did not seem to change the gene expression of the tight junction-associated protein ZO-1, pore-forming claudin-2, or the barrier-forming claudin-3 (Figure 7), two major claudins in the intestinal epithelium (Günzel and Yu, 2013). Even after 1 day of TNFα incubation no obvious changes on the protein expression pattern or localization of ZO-1 or villin in intestinal organoids could be observed (Figures 4A, 8). This is in contrast to other studies, where intestinal inflammation led to a more permeable epithelial barrier measured as increase in tissue conductance in Ussing chamber experiments (Steidle et al., 2013; Ballout and Diener, 2019) either by reducing tightening barrier proteins, e.g., claudin-3 or claudin-4, or by enhancing pore-forming proteins like claudin-2 (Zeissig et al., 2007; Barmeyer et al., 2017). Interestingly, Gleeson et al. (2020) could also not find any difference in the claudin-2 expression in human intestinal organoids after 72 h incubation with 10 ng/mL TNFα and IFN-γ. Hence, our finding that TNFα evoked organoid swelling (Figure 3; Supplementary Video S1) cannot be explained by a disrupted epithelial barrier and a passive influx of water. The permeability of the epithelial barrier might underlie a more complex regulation during inflammation. There might be concentration- and/or time-dependent cytokine effects or modulation by other cells located in the intestinal wall, e.g., immune cells or enteric neurons, known to release mediators enhancing the paracellular permeability under inflammatory conditions (Keita and Söderholm, 2010; van Spaendonk et al., 2017). Such a concentration and time dependency by TNFα on intestinal organoids has also been observed for other components of the epithelium, such as Muc2, a marker for goblet cells. Contradictory results show that 10 ng/mL to 30 ng/mL TNFα induced either an up- or downregulation of Muc2 gene or protein expression in murine or human stem cell derived intestinal organoids over a time period of 3 h–96 h (Li et al., 2018; Onozato et al., 2021; Saito et al., 2021), whereas in the present study 100 ng/mL TNFα for 5 days reduced the Muc2 expression in intestinal organoids. This might result in a thinner mucus layer and reduce barrier integrity with higher predisposition for the development of intestinal inflammation as shown in DSS colitis or Muc2^−/−^ mice (Petersson et al., 2011).

As stated in the introduction, the intestinal secretion is dominated by the transport of Cl^−^, involving different Cl^−^ transporters and channels (e.g., NKCC1, CFTR, Ca^2+^-activated anion channels). This secretory process is regulated by the enteric nervous system and the release of classical neurotransmitters as acetylcholine or VIP, which stimulate a second messenger cascade (Ca^2+^ or cAMP, respectively) via distinct G protein-coupled receptors (G_q_ for cholinergic M_1_/M_3_ receptors or G_s_ for VIP receptor) (Böhme et al., 1991; Lindqvist et al., 1998; Barrett and Keely, 2000). In the present experiments, the acetylcholine derivate, carbachol, evoked an increase in cytosolic Ca^2+^ in intestinal organoids, measured as increase in the fura-2 ratio (Table 3; Figure 9). That intestinal organoids express functional cholinergic receptors has also been proven by Donowitz’s group, where acetylcholine induced an anion secretion in monolayers derived from murine jejunal organoids (Johnson et al., 2020). Interestingly, the increase in the fura-2 ratio induced by carbachol seems to intensify with the age of organoids. Whether this might be caused by an upregulation of muscarinic receptors or components of the signaling cascade involved, remains unclear at the moment. However, the basal fura-2 ratio, i.e., the basal cytosolic Ca^2+^ concentration, did not show such a time-dependent drift (Table 3).

As TNFα is a prominent proinflammatory cytokine involved in intestinal inflammation, where diarrhea is one of the main symptoms (McKay and Baird, 1999; Martínez-Augustin et al., 2009; Keely and Barrett, 2022), we investigated the direct effects of this cytokine on epithelial cells from a small intestinal murine organoid model. Incubation with TNFα (100 ng/mL) for 24 h resulted in a progressive “organoid swelling” probably due to a higher net secretion of electrolytes and water into the organoid lumen (Supplementary Video S1; Figures 3, 5). This is in accordance with previous results from our lab, where 100 ng/mL TNFα induced an increase in short-circuit current (I_sc_) indicating an enhanced anion secretion in mucosa-submucosa preparations of rat distal colon (Ballout and Diener, 2019). The exact mechanisms underlying the cytokine-induced secretion across intestinal epithelium are still unclear. One explanation might be a higher NKCC1 expression after TNFα treatment (Figure 7). This is consistent with the finding that bumetanide, an inhibitor of the NKCC1, reduced the TNFα-induced organoid swelling by approximately 50% (Figure 6). The impact of TNFα on NKCC1 expression has also been reported in other studies, where TNFα and IL-1β upregulate the expression of NKCC1 in human umbilical vein endothelial cells (Topper et al., 1997) and in rat astrocytes (Huang et al., 2014).

The results from these swelling experiments raise the question if TNFα might affect Cl^−^ secretion via Ca^2+^, a second messenger centrally involved in this signal pathway (Barrett and Keely, 2000). As stated in the introduction, both downregulation (Kachur et al., 1995) or complete preservation of Ca^2+^-induced secretion (after removal of the submucosal plexus; see also Sánchez de Medina et al., 2002) have been reported in the literature. Ca^2+^ can be released from intracellular stores via the PLC/IP_3_ pathway followed by a Ca^2+^ influx from the extracellular space via store-operated Ca^2+^ channels (for a detailed review about Ca^2+^ signaling see Clapham, 2007). Ca^2+^ imaging experiments revealed time-dependent effects of TNFα incubation including changes in the basal Ca^2+^ levels (higher at 1 day, unaltered at 3 days, and lower at 5 days; see Table 3). Preincubation for 3 days with the cytokine enhanced the carbachol-induced rise in the cytosolic Ca^2+^ concentration (Table 3). This probably reflects a facilitation of Ca^2+^ influx from the extracellular side rather than a Ca^2+^ release from intracellular stores, as this effect was abolished in Ca^2+^-free buffer or in the presence of La^3+^, a broad blocker of nonselective cation channels (Table 4; Figure 9). Plausible candidates for (nonselective) Ca^2+^-permeable cation channels, which might be involved in this facilitation, are members of the transient receptor potential (TRP) superfamily. Paradoxically, La^3+^ induced a transient increase in the cytosolic Ca^2+^ concentration (Figure 9). At first glance, this seems to contradict the assumption that lanthanum blocks Ca^2+^-permeable cation channels in the intestinal organoids. However, several members of the TRP superfamily (e.g., TRPC1, TRPC5, TRPV1, TRPV6) exert a dose-dependent activation or potentiation by lanthanides (Jung et al., 2003; Bouron et al., 2014). Nevertheless, after the decay of the paradox La^3+^-induced rise in cytosolic Ca^2+^, the lanthanide nearly suppressed the effect of muscarinic receptor stimulation indicating a blockade of Ca^2+^ channels in the plasma membrane.

In addition to Ca^2+^, intestinal Cl^−^ secretion is also controlled by the cAMP second messenger system. For example, PGE_2_ (when acting on EP2 or EP4 receptors) and VIP activate G_s_ protein-coupled receptors, followed by increased cAMP production via adenylate cyclases, stimulation of protein kinase A, and opening of apical CFTR channels resulting in a Cl^−^ secretion into the gut lumen (Boige et al., 1984). In human jejunal organoids, PGE_2_ and VIP led to an even more effective organoid swelling than acetylcholine (Fujii et al., 2016). However, in the present experiments blockade of prostaglandin synthesis with indometacin did not inhibit TNFα-induced swelling (Figure 6) and also the forskolin-induced secretion did not differ from the untreated controls suggesting that cAMP signaling may only play a minor role in the observed responses.

Nevertheless, diarrhea under inflammatory conditions can not only be explained by an enhanced Cl^−^ secretion, but also by a reduced Na^+^ absorption (also leading to a higher net secretion) and increased leak flux via the impaired epithelial barrier. During IBD a diminished expression or activity of Na^+^-dependent transporters, e.g., the Na^+^/K^+^ pump or epithelial Na^+^ channels (ENaCs), have been reported in previous studies (Amasheh et al., 2004; Martínez-Augustin et al., 2009; Magalhães et al., 2016). If and how these transporters play a role in intestinal organoid secretion after cytokine treatment has to be elucidated in further experiments.

## Conclusion, limitations and outlook of the present study

This study focused on the direct (long-term) effects of TNFα on intestinal epithelium function using a murine small intestinal organoid model. We observed that NKCC1 is centrally involved in the TNFα-induced organoid swelling due to bumetanide sensitivity and enhanced expression of the transporter. Furthermore, TNFα seems to affect only the Ca^2+^ but not the cAMP-dependent Cl^−^ secretion, probably by modulating La^3+^-sensitive Ca^2+^-permeable channels located in the cell membrane rather than intracellular Ca^2+^ stores. Albeit we could show that organoids are a useful tool to study secretory effects under inflammatory conditions, it has to be kept in mind that TNFα effects are more extensive (e.g., apoptosis or proliferation) and that other cell types in the intestinal epithelium (e.g., goblet cells) can be affected by the cytokine. Furthermore, intestinal inflammation is a complex interplay between the epithelium, the enteric nervous system, and the immune system driven by a variety of neurotransmitters and other (immune) mediators. This explains, for example, the resistance of TNFα-induced secretion to indometacin (Figure 6), although data from the literature clearly point at a central role of eicosanoids in this process (Schmitz et al., 1996; Schmitz et al., 1999). However, the most prominent production sites for prostaglandins are cells located in the subepithelial tissue (Craven and DeRubertis, 1986), which is missing in the organoid culture. Hence, combining intestinal organoids with, e.g., fibroblasts or monocytes/macrophages would represent cell-cell-interactions under inflammatory conditions in a more detailed manner. Further limitations of the present study lay in the time points selected for qPCR analysis, and it might be possible that changes in gene expression could have been observed at earlier (or later) time points. In addition, expression changes at the protein level are clearly necessary for a detailed analysis of the actions of TNFα on the epithelium. These might either be performed by Western blots for a few selected proteins or—in our view a more promising option—by an untargeted analysis of the composition of the organoids after cytokine exposure by mass spectrometry imaging. Such an untargeted approach would not only allow to study the regulation of a whole range of proteins by TNFα (Huber et al., 2018), but also of changes in lipid composition of the epithelial cells (Anschütz et al., 2023) and for which a method is currently being established in ongoing experiments.

## Data Availability

The original contributions presented in the study are included in the article/Supplementary Material, further inquiries can be directed to the corresponding author.
